# Factors Related to Job Satisfaction Among Moroccan Nurses: A Cross-Sectional Secondary Data Analysis

**DOI:** 10.7759/cureus.89078

**Published:** 2025-07-30

**Authors:** Wafaa Al Hassani, Youness El Achhab, Hafid Hachri, Abdelali Alaoui Belghiti, Chakib Nejjari

**Affiliations:** 1 Nursing Sciences, Euromed University of Fes, Fez, MAR; 2 Biological Sciences, Regional Center for Education and Training Careers Fez-Meknes, Fez, MAR; 3 Epidemiology and Public Health, Country Office of Morocco, World Health Organization, Rabat, MAR; 4 Health Policy, Hospital Management, and Health Systems Development, Euromed University of Fes, Rabat, MAR; 5 Epidemiology and Public Health, Euromed Research Center, Euromed University of Fes, Fez, MAR; 6 Epidemiology and Public Health, Faculty of Medicine, Pharmacy and Dentistry, Sidi Mohammed Ben Abdellah University, Fez, MAR

**Keywords:** correlates, job satisfaction, morocco, nurses, regression analysis

## Abstract

Background

Nurses’ job satisfaction directly affects the quality of patient care and organizational performance, yet little is known about its determinants in Morocco. This study assessed overall job satisfaction levels and examined how key demographic and workplace factors influence these levels.

Methods and materials

A secondary analysis was conducted on the 2018 Ministry of Health national survey of healthcare workers. The sample consisted of 842 nurses from 160 public institutions, selected through a stratified two-stage cluster design. Job satisfaction was measured with a 25‑item, seven-domain scale (total possible score=25-125; Cronbach’s alpha=0.86). Descriptive statistics, Mann-Whitney U and Kruskal-Wallis tests, and multiple linear regression were used to explore associations between job satisfaction scores and demographic variables (age, gender, and years of experience) as well as workplace characteristics (institution type and geographic region).

Results

The mean total job satisfaction score was 71.5±12.7, indicating a moderate level overall. No significant differences were observed by gender or years of experience. Nurses aged 40-50 years scored higher than those aged <40 or >50 years (p<0.05). Health center nurses reported lower satisfaction than hospital or other facility nurses (p<0.01). Those in the north‑central region were less satisfied than their peers in the western and eastern southern regions (p<0.01). In multivariable analysis, only institution type (β=2.75, p<0.01) and region (β=1.24, p=0.03) remained significant predictors, although the adjusted R² was modest (0.025).

Conclusion

Moroccan nurses exhibit moderate job satisfaction overall, with notably lower scores in rural primary healthcare centers and less resourced regions. Addressing workplace conditions through infrastructure upgrades, incentive packages, staffing support, and professional development opportunities could enhance satisfaction and retention, ultimately improving health service equity and quality.

## Introduction

Globally, the nursing workforce has become more diverse, and challenges persist, including aligning workforce diversity with the population, addressing compensation and opportunities, and exploring the roles of nurses in new practice settings [1-3]. It is crucial to focus on improving the quality of nursing care, especially since nurses make up the largest proportion of healthcare professionals. Additionally, they play a critical role in patient care and safety through continuous services and interactions [4]. The quality of nursing care, reflected in job satisfaction, has become a significant concern for healthcare organizations [5-7].

The topic of job satisfaction among health professionals has been a subject of global interest for many years. Nurses' job satisfaction is a crucial factor in the recruitment and retention of nursing staff in healthcare delivery systems [8,9]. The level of job satisfaction can depend on how highly an employee values the rewards and benefits associated with their job [10]. In nursing research, a variety of definitions of nurses' job satisfaction have been proposed by researchers [11]. However, there is no clear consensus on the definition of nurses' job satisfaction. In their concept analysis study, Liu et al. aimed to describe and clarify the multifaceted nature of job satisfaction [11]. They emphasized that the main attributes of job satisfaction include meeting desired needs at work, experiencing positive emotions toward working conditions, and feeling a sense of value or fairness in one's job.

Individual and organizational factors of nurses' job satisfaction have been described in several studies [12-16]. Factors related to individual predispositions, such as age, gender, marital status, years of experience, and level of education, have been found to be associated with or have an impact on nurses' job satisfaction. Organizational productivity and quality patient care are impacted by nurses' job satisfaction [15-19]. Satisfied nurses perform their duties efficiently, boost productivity, and provide high-quality care. In contrast, dissatisfied nurses often exhibit passive attitudes, which can lead to turnover, conflict, and absenteeism, eventually resulting in decreased work efficiency. Low income, career development, and heavy workload were reported to be major factors associated with lower nurses’ job satisfaction [20,21].

The healthcare system of the Moroccan people faces several challenges [22]. The Moroccan Ministry of Health (MoH) has aligned with World Health Organization recommendations [23] by investing in human resources for health through various national programs and studies to address issues, such as coverage, shortages, continuing education, motivation, and satisfaction (Report: MoH. Universal health coverage in Morocco: challenges for human resources; 2017; Report: MoH. Continuing training strategy for health professionals; 2017; and Report: MoH. Study of satisfaction/motivation among health professionals; 2018) [24]. Job satisfaction in healthcare institutions was first evaluated by the MoH among health professionals (Report: MoH. Study of satisfaction/motivation among health professionals; 2018). The main result of this study was that 64% of all participants were dissatisfied. Recently, two studies have examined nurses' job satisfaction, finding it to be at a moderate to low level [25,26]. A broad search of major international and regional scientific databases from inception to March 31, 2025, using combinations of terms "nurse," "job satisfaction," "determinant OR correlate," and "Morocco," identified no prior studies examining the correlates of nurses’ job satisfaction in Morocco. This study presents a secondary analysis of data from the MoH's national study (Report: MoH. Study of satisfaction/motivation among health professionals; 2018). This study aimed to investigate nurses' job satisfaction and its correlates based on a validated instrument [27].

## Materials and methods

Data collection and sampling

This study is a cross-sectional secondary data analysis using survey data from a national study conducted by the Ministry of Health to evaluate the satisfaction and motivation of health professionals in the public sector (Report: MoH. Study of satisfaction/motivation among health professionals; 2018). The procedure surrounding the survey, as well as the sampling approach and data collection, has been previously described [27]. Briefly, 2,122 survey questionnaires were completed via face-to-face interviews in 2018 by health professionals working in 160 health institutions in all 12 Moroccan regions. This study analyzed data from 842 nurses who participated in this survey.

Measure

Job satisfaction was assessed using the job satisfaction scale developed and validated in the conducted national survey [27]. This scale comprises 25 items across seven domains: career development (six items), working conditions (four items), social support (four items), role clarity (three items), workload (four items), remuneration (two items), and institutional relationships (two items). All items are rated on a five-point Likert-type scale, ranging from "strongly disagree" (1) to "strongly agree" (5). The item scores were averaged for each domain. The 25-item scale yields a theoretical total score from 25 to 125. Following the convention used in the scale-validation paper and splitting the continuum into three equal intervals, we interpret totals as follows: low satisfaction (25-58), moderate satisfaction (59-92), and high satisfaction (93-125) [27].

In this study, the Cronbach’s alpha reliability coefficient was evaluated, and the scores for each item were as follows: career development (0.86), working conditions (0.77), social support (0.74), role clarity (0.80), workload (0.76), remuneration (0.84), and institutional relationships (0.75). The overall Cronbach’s alpha coefficient for the scale used in this study was 0.86, and the model demonstrated satisfactory fit indices, with root mean square error of approximation (RMSEA)=0.05, χ²/df=6.4, Tucker-Lewis Index (TLI)=0.92, and Comparative Fit Index (CFI)=0.93.

In this study, demographic characteristics included personal (age and gender) and job-related variables (institution, years of work experience, and region). Morocco is divided into 12 regions. We redistributed the data to three zones. The western zone, encompassing the Casablanca-Settat region and Rabat-Salé-Kénitra region, is the most populated and economically advanced area.

Data analysis

All analyses were performed using Jamovi software version 2.3.28 (Sydney, Australia: Jamovi Project Ptv Ltd.). The dataset used for analysis did not contain any missing values across the variables included in the study. Descriptive analysis was performed for data exploration. For analytic parsimony, the 12 administrative regions were grouped into three zones that share similar sociodemographic and health-system characteristics as follows: (1) West (Casablanca-Settat and Rabat-Salé-Kénitra): densely populated, urban, and economically advanced; (2) North-Central (Tangier-Tétouan-Al Hoceïma, Fès-Meknès, Marrakesh-Safi, Béni Mellal-Khénifra): mixed urban-rural with intermediate resources; and (3) East and South (the remaining six regions): sparsely populated, predominantly rural.

In this analysis, the job satisfaction score was treated as a continuous variable, consistent with other studies [16,28,29]. The normality of the data was tested using the Kolmogorov-Smirnov test. Given the non-normal distribution of the data, comparisons of mean total job satisfaction and its subdomain scores across demographic variables were conducted using the Mann-Whitney U test and Kruskal-Wallis H test for categorical variables with two, three, or more categories, respectively. Spearman's rank correlation coefficients were calculated to evaluate the relationship between a dependent variable (total job satisfaction) and seven subdomains of job satisfaction.

## Results

Sociodemographic characteristics of the study participants

A total of 842 nurses, including 279 males (33.2%), participated in this study. Nearly half of the participants were under 40 years of age (45.6%). The majority worked in hospitals (59.7%), while 27.0% were employed at health centers. Regarding years of experience, 64.8% had been working for 10 years or more. The participants predominantly lived in the North-Central (38.2%) and East-Southern (36.9%) regions of Morocco (Table 1).

**Table 1:** Sociodemographic characteristics of the participants. *West: Casablanca-settat and Rabat-Salé-Kénitra. **North-Central: Tangier-Tetouan-Al Hoceima, Fès-Meknès, Marrakesh-Safi, and Béni Mellal-Khenifra. ***East and South: Dakhla-Oued Ed-Dahab, Laâyoune-Sakia El Hamra, Guelmim-Oued Noun, Souss-Massa, Drâa-Tafilalet, and Oriental.

Characteristics	Category	Frequency (n)	Percentage (%)
Gender	Male	279	33.2
	Female	561	66.8
Age (years)	<40	383	45.6
	40-50	194	23.1
	>50	263	31.3
Institution	Health center	227	27.0
	Hospital	503	59.7
	Other	112	13.3
Working years	≤10	296	35.2
	>10	544	64.8
Region	West*	209	24.8
	North-Central**	322	38.2
	East and South***	311	36.9

Level of job satisfaction

The total job satisfaction scores of the 842 nurses, along with the scores on the seven subscales, are presented in Table 2. The mean scores for the career development (mean: 16.3, median: 16), social support (mean: 11.1, median: 11), workload (mean: 9.09, median: 9), and remuneration (mean: 8.26, median: 8) subscales were slightly greater than their median scores. However, the mean scores for the working condition (mean: 7.86, median: 8), role clarity (mean: 9.93, median: 10), and institution relationship (mean: 8.92, median: 9) subscales were lower than their respective median scores. The mean total job satisfaction score was 71.5±12.7, and half of the nurses had a mean score above the median of 71. The majority of the nurses scoring above the median were female (66.6%), under 40 years of age (45%), and from the western region (45.7%).

**Table 2:** Descriptive statistics of the job satisfaction subscales.

Job satisfaction with	Number of item	Score range	Median	Mean±SD
Career development	6	6-30	16	16.3±5.08
Working condition	4	4-20	8	7.86±2.72
Social support	4	4-20	11	11.1±3.53
Role clarity	3	3-15	10	9.93±2.86
Workload	4	4-18	9	9.09±3.30
Remuneration	2	2-10	8	8.26±1.97
Institutional relationship	2	2-10	9	8.92±1.50
Total job satisfaction	25	34-113	71	71.5±12.7

Factors associated with job satisfaction

Table 3 presents the sociodemographic differences in mean job satisfaction and subscale scores, as well as the results of the multiple linear regression analysis. Female nurses scored significantly higher than their male colleagues on the role clarity (mean: 10.11, SD: 2.91) and institutional relationship (mean: 9.06, SD: 1.35) subscales (p<0.05). Nurses in the 40- to 50-year-old age group scored significantly higher in terms of career development, working conditions, social support, role clarity, workload, and remuneration. Nurses with more than 10 years of experience scored significantly higher on the social support (mean: 11.64, SD: 3.45), role clarity (mean: 10.22, SD: 2.94), and remuneration (mean: 8.40, SD: 1.88) subscales than did those with 10 or fewer years of experience. Nurses in the eastern and southern regions scored significantly higher on the career development (mean: 17.65, SD: 4.22), working condition (mean: 8.65, SD: 2.51), and workload (mean: 9.43, SD: 2.37) subscales than did their colleagues in other regions. Except for age group and gender, institutional relationship scores did not differ across the other five subscales between institutions, working years, and region groups.

According to the total job satisfaction scores, there were no statistically significant differences in the mean scores between gender and working years. However, nurses aged 40-50 years (mean score: 73.30, SD: 15.13) had significantly greater total job satisfaction scores than did those aged older than 50 years (mean score: 70.22, SD: 11.22) and those aged younger than 40 years (mean score: 71.46, SD: 12.25). Health center workers had significantly lower mean total job satisfaction scores (mean: 70.49, SD: 11.48) than did those working in hospitals (mean: 71.18, SD: 13.58) or other institutions (mean: 76.67, SD: 14.78). Nurses in the north-central region had significantly lower mean total job satisfaction scores (mean: 68.59, SD: 11.37) than did those in the western region (mean: 72.46, SD: 17.34) and the eastern and southern regions (mean: 73.86, SD: 9.31).

**Table 3:** Sociodemographic differences in mean job satisfaction and subscale scores and multiple linear regression analysis (F {3,836}=8.11, p<0.001). *P<0.05 denotes statistical significance. **P<0.01 denotes high statistical significance.

Variables	Number	Career development	Working condition	Social support	Role clarity	Workload	Remuneration	Institutional relationship	Total job satisfaction	Multiple linear regression β (SE)
Gender										
Male	279	16.47±5.01	7.71±2.58	11.46±3.35	9.57±2.76*	9.33±3.13	8.28±1.95	8.66±1.72*	71.47±12.89	-
Female	561	16.20±5.12	7.92±2.79	10.99±3.60	10.11±2.91	8.97±3.39	8.26±1.99	9.06±1.35	71.51±12.63	-
Age (years)										
<40	383	16.56±4.89*	8.38±2.68**	10.61±3.48**	9.62±2.72*	9.27±2.99**	8.03±2.06*	8.98±1.35**	71.46±12.25*	-
40-50	194	16.93±5.19	7.44±2.96	12.02±3.50	10.31±2.97	9.63±3.85	8.55±1.72	8.41±1.81	73.30±15.13	-0.59 (0.51)
>50	263	15.41±5.19	7.38±2.47	11.28±3.49	10.11±2.96	8.42±3.22	8.40±1.98	9.22±1.35	70.22±11.22	-
Institution										
Health center	227	16.30±4.68	7.86±2.63*	11.02±3.44**	9.70±2.84*	8.40±2.85**	8.25±1.98	8.96±1.41	70.49±11.48**	-
Hospital	503	16.15±5.47	7.51±2.60	10.49±3.42	10.21±2.99	9.62±3.71	8.34±1.97	8.87±1.66	71.18±13.58	-
Other	112	16.59±5.95	8.52±3.25	12.96±3.53	10.39±2.66	11.14±3.33	8.18±1.96	8.88±1.51	76.67±14.78	2.75 (0.61)**
Working years										
≤10	296	16.70±4.91*	8.75±2.65**	10.24±3.50**	9.41±2.65**	9.32±2.97*	8.02±2.11*	8.82±1.58	71.27±12.52	-
>10	544	16.07±5.17	7.36±2.64	11.64±3.45	10.22±2.94	8.96±3.47	8.40±1.88	8.98±1.44	71.62±12.82	-
Region										
West	209	16.58±6.30**	6.99±3.01**	11.60±4.12*	10.57±3.18**	9.27±4.31**	8.71±1.95**	8.73±2.05	72.46±17.34**	-
North-Central	322	14.80±4.54	7.65±2.52	10.81±3.51	9.72±2.89	8.65±3.29	7.98±1.98	8.98±1.23	68.59±11.37	-
East and south	311	17.65±4.22	8.65±2.51	11.16±3.05	9.72±2.54	9.43±2.37	8.25±1.92	9.00±1.28	73.86±9.31	1.24 (0.57)*

After including all significant associations from the univariate analyses in the multiple linear regression analysis, only institution (beta coefficient=2.75, p<0.01) and region (beta coefficient=1.24, p=0.03) remained significant predictors of the mean total job satisfaction score. The model had an adjusted R² of 0.025. The regression model was highly significant, F (3,836)=8.11, p<0.001.

Significant (p<0.001) correlations ranging from strong to weak were found between total job satisfaction and all seven job satisfaction subscales, as follows: career development (r=0.730), working conditions (r=0.380), social support (r=0.677), role clarity (r=0.579), workload (r=0.693), remuneration (r=0.228), and institutional relationships (r=0.243) (Table 4). A robust correlation was found between total job satisfaction and career development (r=0.730), while the weakest correlation was between remuneration and total job satisfaction (r=0.228).

**Table 4:** Spearman’s correlation coefficient of job satisfaction subscales. *P<0.05 denotes statistical significance. **P<0.01 denotes high statistical significance. ***P<0.001 denotes very high statistical significance.

Variables	Career development	Working condition	Social support	Role clarity	Workload	Remuneration	Institutional relationship	Total job satisfaction
Career development	1	-	-	-	-	-	-	-
Working condition	0.282***	1	-	-	-	-	-	-
Social support	0.319***	0.072*	1	-	-	-	-	-
Role clarity	0.219***	- 0.003	0.362***	1	-	-	-	-
Workload	0.391***	0.239***	0.432***	0.376***	1	-	-	-
Remuneration	0.022	- 0.072*	0.116***	0.124***	0.001	1	-	-
Institutional relationship	0.013	0.064	0.097**	0.230***	0.048	0.254***	1	-
Total job satisfaction	0.730***	0.380***	0.677***	0.579***	0.693***	0.228***	0.243***	1

## Discussion

This study examined the job satisfaction of 842 nurses in Morocco using a representative nationwide sample of 160 health institutions and explored the factors influencing their satisfaction within the work environment. On average, half of the nurses achieved a mean score higher than the median score of 71, indicating a moderate level of job satisfaction. These results were slightly consistent with studies conducted in Ethiopia, where the mean satisfaction level of nurses was 54% [29], and in Saudi primary healthcare centers and hospitals, where the mean satisfaction levels were 58% [30] and 48%, respectively [31]. In Italian public hospitals, nurses' job satisfaction is at a neutral level [32]. In contrast, a study in Jordan found that nurses were moderately satisfied with their work [33]. However, the job satisfaction levels were higher than those reported in studies from Iran (28%) [16] and Chile (21%) [34], as well as in Ireland, where a low level of job satisfaction among public health nurses was highlighted [35]. In contrast, higher job satisfaction among nurses was reported among primary care nurse practitioners in six states in the United States (90%) [36]. The differences in nurses’ job satisfaction levels could be attributed to socioeconomic and organizational factors, as well as variations in study timing and the tools used.

According to the factors associated with job satisfaction, this study revealed that nurses practicing in primary health centers had significantly lower mean total job satisfaction scores and scores on most of the job satisfaction subscales than those working in hospitals or other institutions. As workplace environments, primary health centers differ from hospitals, particularly in terms of their structure. Additionally, hospitals are located in cities, while rural areas are primarily characterized by primary health centers. Workplace structures can promote healthier nurses, reduce stress, and enhance job satisfaction [37]. A satisfying work environment for nurses is related to structural and psychological empowerment in the workplace, which in turn leads to positive retention outcomes such as job satisfaction [38].

Gender and years of experience show conflicting relationships with job satisfaction [39]. Additionally, there is no consensus in the literature regarding age as a determining factor for job satisfaction. The opportunities for higher salaries, greater job stability, and improved quality of life have contributed to the migration of nursing professionals. Therefore, working in a region that offers these opportunities would improve job satisfaction. Several studies reinforce the view that nurses’ job satisfaction is related to regional opportunities [40-42].

Despite the statistical significance of region and institution, the multiple linear regression model explained only a small share of the variability in job‑satisfaction scores. In practical terms, this means that regional location and workplace setting together account for roughly 2.5% of the observed differences, leaving the vast majority of variance unexplained. Previous research suggests that organizational culture, leadership style, workload intensity, interpersonal relationships, and exposure to workplace violence are among the psychosocial and environmental factors that more strongly influence nurse satisfaction but were not captured in the present secondary dataset [12-16]. Consequently, our findings should be interpreted cautiously as follows: while regional and institutional disparities are real, their effect size is modest, and any policy response must also address the broader organizational and individual determinants highlighted in the literature.

Limitations

This study has several limitations that should be acknowledged. First, the use of secondary data restricted the range of variables available for analysis. Important determinants of job satisfaction, such as marital status, educational level, organizational culture, workload intensity, and exposure to workplace violence, were not captured, which limited the scope of explanatory factors. Second, although the original survey used a stratified two-stage cluster design with a high response rate, participation was voluntary, and data were collected on a single workday at each facility. Nurses who were absent or declined participation may differ systematically in their satisfaction, introducing potential selection bias that we cannot quantify. Third, because the research team had no control over the original data collection process, possible measurement inconsistencies could not be assessed or corrected. Finally, the cross-sectional nature of the study prevents causal inference. Future research should employ prospective or mixed-method designs, document non-respondent characteristics, and include a broader range of individual, organizational, and contextual variables to provide a more comprehensive understanding of nurse job satisfaction in Morocco.

## Conclusions

This nationwide analysis shows that Moroccan nurses report moderate overall job satisfaction, with markedly lower scores in rural primary healthcare centers. These disparities mirror patterns seen in other middle‑income countries and suggest structural gaps within the national health system. Targeted improvements in infrastructure, incentives, staffing, and professional development are therefore warranted to boost retention and care quality. While the secondary, cross‑sectional design limits causal inference, our findings provide actionable evidence to guide future workforce policies and longitudinal research.
